# Dose-Dependent Differential Effect of Neurotrophic Factors on In Vitro and In Vivo Regeneration of Motor and Sensory Neurons

**DOI:** 10.1155/2016/4969523

**Published:** 2016-10-27

**Authors:** Daniel Santos, Francisco Gonzalez-Perez, Xavier Navarro, Jaume del Valle

**Affiliations:** Institute of Neurosciences, Department of Cell Biology, Physiology and Immunology, Universitat Autònoma de Barcelona and Centro de Investigación Biomédica en Red sobre Enfermedades Neurodegenerativas (CIBERNED), Bellaterra, Spain

## Abstract

Although peripheral axons can regenerate after nerve transection and repair, functional recovery is usually poor due to inaccurate reinnervation. Neurotrophic factors promote directional guidance to regenerating axons and their selective application may help to improve functional recovery. Hence, we have characterized in organotypic cultures of spinal cord and dorsal root ganglia the effect of GDNF, FGF-2, NGF, NT-3, and BDNF at different concentrations on motor and sensory neurite outgrowth. In vitro results show that GDNF and FGF-2 enhanced both motor and sensory neurite outgrowth, NGF and NT-3 were the most selective to enhance sensory neurite outgrowth, and high doses of BDNF selectively enhanced motor neurite outgrowth. Then, NGF, NT-3, and BDNF (as the most selective factors) were delivered in a collagen matrix within a silicone tube to repair the severed sciatic nerve of rats. Quantification of Fluorogold retrolabeled neurons showed that NGF and NT-3 did not show preferential effect on sensory regeneration whereas BDNF preferentially promoted motor axons regeneration. Therefore, the selective effects of NGF and NT-3 shown in vitro are lost when they are applied in vivo, but a high dose of BDNF is able to selectively enhance motor neuron regeneration both in vitro and in vivo.

## 1. Introduction

After peripheral nerve injury, transected axons can regenerate and reinnervate target organs. However, reinnervation of distal organs and functional recovery are often deficient because random regeneration of axons results in aberrant target reinnervation [1]. Thus, specificity of reinnervation is a key issue to improve functional recovery after peripheral nerve injuries.

Although some studies described that motor axons preferentially reinnervate muscular pathways [2], this accuracy is compromised when optimal conditions such as pure muscular and cutaneous branches, matching distal nerve caliber, and short separation between nerve stumps are not met [3, 4]. It has been suggested that although motor or sensory neurons tend to regenerate through their original pathway, axons sense the levels of trophic factors in distal branches and then grow towards the target that offers more trophic support [5]. In fact, some authors argue that the key point for preferential attraction of axons to their adequate targets is the expression of trophic factors by their own target organ and the distal stump [2, 3].

After nerve injury, in order to support neuronal survival and enhance regeneration, motoneurons in spinal cord (SC) and sensory neurons in dorsal root ganglia (DRG) synthesize and secrete neurotrophic factors (NTFs). Furthermore, within the proximal and the distal nerve stumps, denervated Schwann cells become growth supportive and secrete several NTF and cytokines that follow different patterns of expression, with an initial upregulation of NGF, BDNF, and GDNF, whereas others, such as NT-3 or CNTF, are downregulated [6]. However, changes in NTF levels are severalfold higher at the lesion area than in the SC or the DRG where the somata of the axotomized neurons are located [7]. Moreover, it has been described that some NTF may influence the direction of regenerating axons on certain models of regeneration [8]. Thus, modifying the regenerative microenvironment is a promising approach to modulate and selectively enhance sensory and motor regeneration.

However, high levels of NTF or a prolonged delivery over time can in fact induce no improvement or even result in deleteriousness in terms of regeneration [9–11], inducing neurotoxicity [12] and endoneurial sprouting and hyperalgesia [13]. Therefore, as low levels of NTF may not reach therapeutical action and high levels may disrupt regeneration, a comparative study of the concentrations and delivery of NTF to specifically improve regeneration of motor or sensory neurons was undertaken. We analyzed the effect of GDNF, FGF-2, NGF, NT-3, and BDNF at different concentrations on motor and sensory axonal regeneration using in vitro and in vivo models.

## 2. Materials and Methods

### 2.1. Ethical Guidelines

Both in vitro and in vivo experimental procedures were approved by the Ethical Committee of the Universitat Autònoma de Barcelona in accordance with the European Communities Council Directive 2010/63/EU. Adult rats were anaesthetized with pentobarbital sodium (40 mg/kg i.p.). P7 and adult rats were euthanized by pentobarbital sodium overdose (200 mg/kg i.p.).

### 2.2. DRG and SC Organotypic Cultures

Organotypic cultures were prepared as previously described [14]. Briefly, collagen I (rat tail, #354236, Corning) diluted in basal Eagle's medium (Gibco) and sodium bicarbonate at 0.3 mg/mL was prepared as control condition. NGF, NT-3, GDNF, and BDNF (Peprotech) were added to achieve concentrations of 5, 10, 50, or 100 ng/mL. As FGF-2 has been reported to work at higher concentrations [15], FGF-2 18 kDa (Peprotech) was prepared at 25, 50, 250, and 500 ng/mL. Finally, 30 *μ*L single drops of the prepared matrices were deposited on poly-d-lysine (1 g/mL, Sigma) coated 24-well multidishes (Iwaki, Asahi Technoglass, Chiba, Japan) and were left to gel for 2 h at 37°C and 5% CO_2_ in the incubator.

SC lumbar segments and lumbar DRG were harvested from 7-day-old Sprague-Dawley rats, placed in 4°C Gey's balanced salt solution (Sigma) enriched with 6 mg/mL glucose and cleaned from blood and meningeal debris. SCs were cut with a McIlwain Tissue Chopper in 350 *μ*m thick slices. SC slices and DRG explants were placed on top of the collagen matrix and covered by a second 30 *μ*L drop of the same solution. After 45 min in the incubator, samples were embedded with 0.5 mL of neurobasal medium (Life Technologies), supplemented with B27 (Life Technologies) and glutamine and penicillin/streptomycin (Sigma). After one day in culture, the medium of SC cultures was removed and changed by a penicillin/streptomycin-free medium. DRG explants were cultured for 2 days and SC slices for 4 days. A detailed description of this protocol has been previously reported [16].

### 2.3. Neurite Outgrowth Analysis

SC and DRG cultures were fixed with 4% paraformaldehyde in PBS for 30 min. Afterwards, SC and DRG samples were incubated for 48 h with primary antibody mouse RT97 (1 : 200, Developmental Studies Hybridoma Bank) at 4°C. After three hours of washing, the sections were incubated with secondary antibody AF594 conjugated donkey anti-mouse (1 : 200, Life Technologies) overnight at 4°C. After two more washes, samples were mounted on slides with Mowiol. Cultures were visualized with an Olympus BX51 fluorescence microscope; images of different areas were taken with a DP50 camera attached to a computer with Cell A software (Olympus) and merged using Adobe Photoshop CS3 (Adobe System). Whole culture images were analyzed with the Neurite-J plug-in [17] for ImageJ software [18] and the number of neurites grown at different distances from the explant was compared between different conditions of the cultures. The area under the curve (AUC) for each group was calculated by the linear trapezoidal method using the amount of neurites every 25 *μ*m.

### 2.4. In Vivo Study of Peripheral Nerve Regeneration

Only the NTFs which showed trophic selectivity for motor and sensory outgrowth were further tested in vivo. Thus, NGF, NT-3, and BDNF at 1, 2, and 10 *μ*g/mL each were added to a 2 mg/mL collagen solution prepared as above. These mixtures were used to fill silicone tubes (8 mm long, 3 mm wide, and 2 mm i.d.) that were maintained vertically for 12 h to promote collagen fibril alignment during gel formation [19]. A collagen matrix without NTF was used in the control group.

Female Sprague-Dawley rats weighing 250–300 g (*n* = 4 group) were anaesthetized, the sciatic nerve was exposed at the midthigh and sectioned 90 mm from the tip of the third toe, and a 6 mm nerve portion distal to the section was resected. The prepared tube was then sutured with 10-0 monofilament sutures to each nerve end leaving a 6 mm gap between nerve stumps. Animals were left to recover on a warming pad and then housed with littermates. They were kept on standard laboratory conditions with a light-dark cycle of 12 : 12 h and ad libitum access to food and tap water. All efforts were made to minimize pain and animal discomfort during surgery and recovery.

### 2.5. Retrograde Labeling and Neuronal Counting

Rats were anesthetized 20 days after operation with pentobarbital sodium; the sciatic nerve was exposed and transected 8 mm distal to the distal end of the silicone tube. Then, the tip of the severed nerve was soaked into 5 *μ*L Fluorogold (FG; 5%; Fluorochrome Inc.) for 1 h in a Vaseline well. After retrieval of the well, saline was flushed to clean remnants of the tracer before suturing the wound in planes. Seven days later, the rats were deeply anesthetized and transcardially perfused with 4% paraformaldehyde in PBS. The lumbar segment (L3–L6) of the SC and L4 and L5 DRG were removed, postfixed in the same fixative solution for 1 h, and transferred to 30% sucrose in PBS. The SC and DRG were cut longitudinally in 40 and 20 *μ*m thick sections, respectively, in a cryostat and mounted on slides. Sections were observed with an Olympus BX51 fluorescence microscope under UV light and the number of labeled neurons was counted in every third section following the fractionator principle [20].

### 2.6. Data Analysis

Data are presented as mean ± SEM. Results were statistically analyzed by using GraphPad Prism (GraphPad Software, USA). Student's *t*-test and one-way ANOVA followed by Bonferroni's post hoc test for comparison between groups were used when applicable. Statistical significance was considered when *p* value was < 0.05.

## 3. Results and Discussion

**Figure 1:**
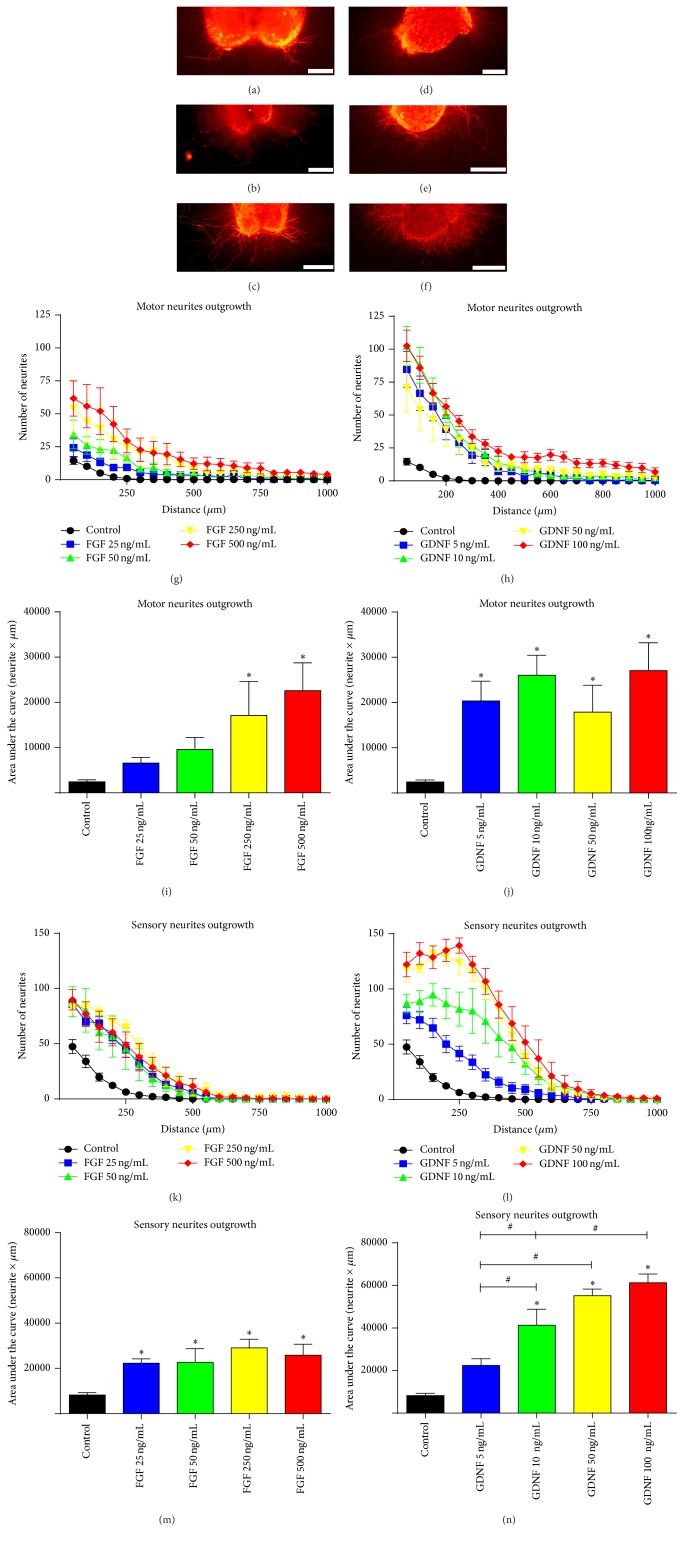
Representative images of RT97 stained neurites from spinal cord slices (a–c) and DRG neurons (d–f) cultured within a 3D collagen matrix alone (a, d) and with addition of 500 ng/mL of FGF-2 (b, e) or 100 ng/mL of GDNF (c, f). Quantification of the number of neurites grown at increasing distance from the spinal cord slices (g, h) and from DRG body (k, l) after the addition of FGF-2 and GDNF. Plots of the quantified AUC from (g), (h), (k), and (l) graphs for motor (i, j) or sensory (m, n) neurite outgrowth. Data expressed as mean ± SEM. ^*∗*^ *p* < 0.05 versus control; ^#^ *p* < 0.05. Scale bar: 100 *μ*m (a) and 200 *μ*m (b–f).

**Figure 2 fig2:**
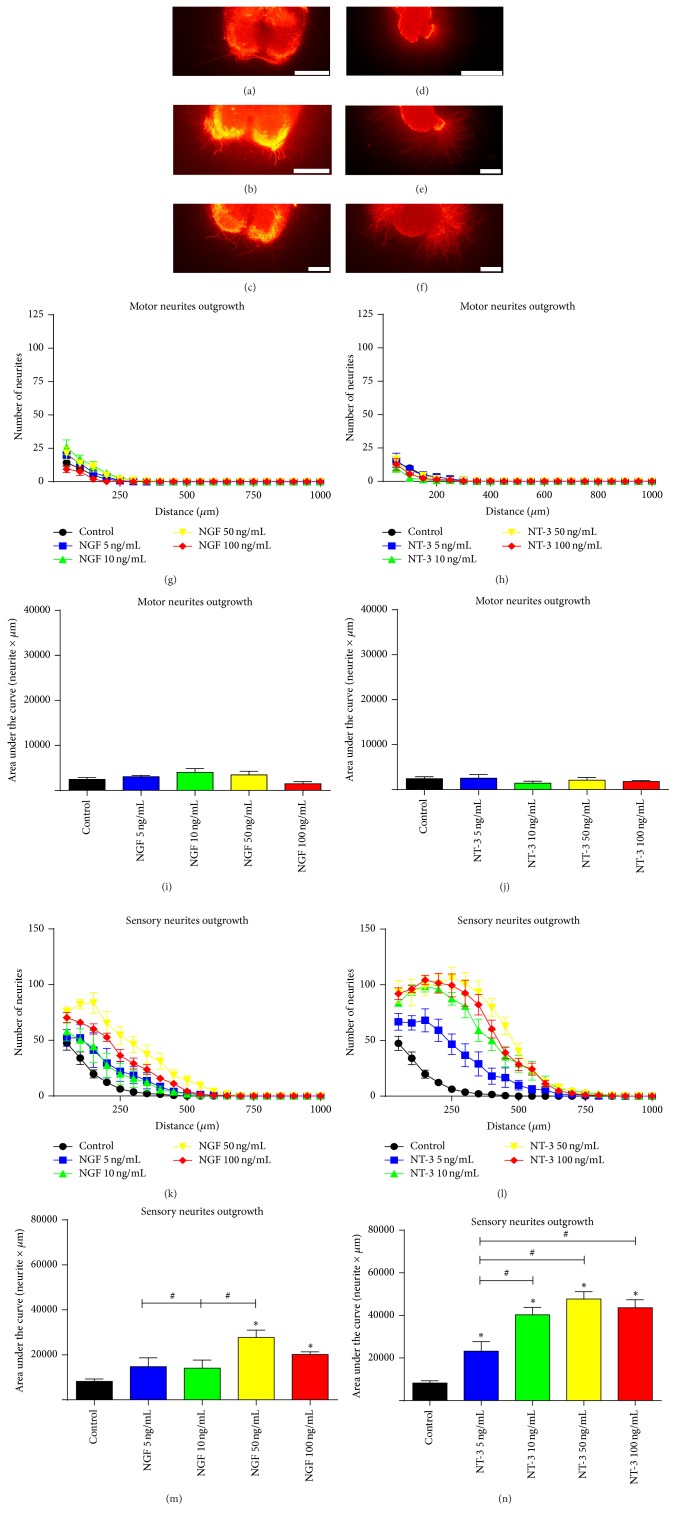
Representative images of RT97 stained neurites from spinal cord slices (a–c) and DRG neurons (d–f) cultured within a 3D collagen matrix alone (a, d) and with addition of 50 ng/mL of NGF (b, e) or NT-3 (c, f). Quantification of the number of neurites grown at increasing distance from the spinal cord slices (g, h) and from DRG body (k, l) after the addition of NGF and NT-3. Plots of the quantified AUC from (g), (h), (k), and (l) graphs for motor (i, j) or sensory (m, n) neurite outgrowth. Data expressed as mean ± SEM. ^*∗*^ *p* < 0.05 versus control; ^#^ *p* < 0.05. Scale bar: 200 *μ*m (a–f).

**Figure 3:**
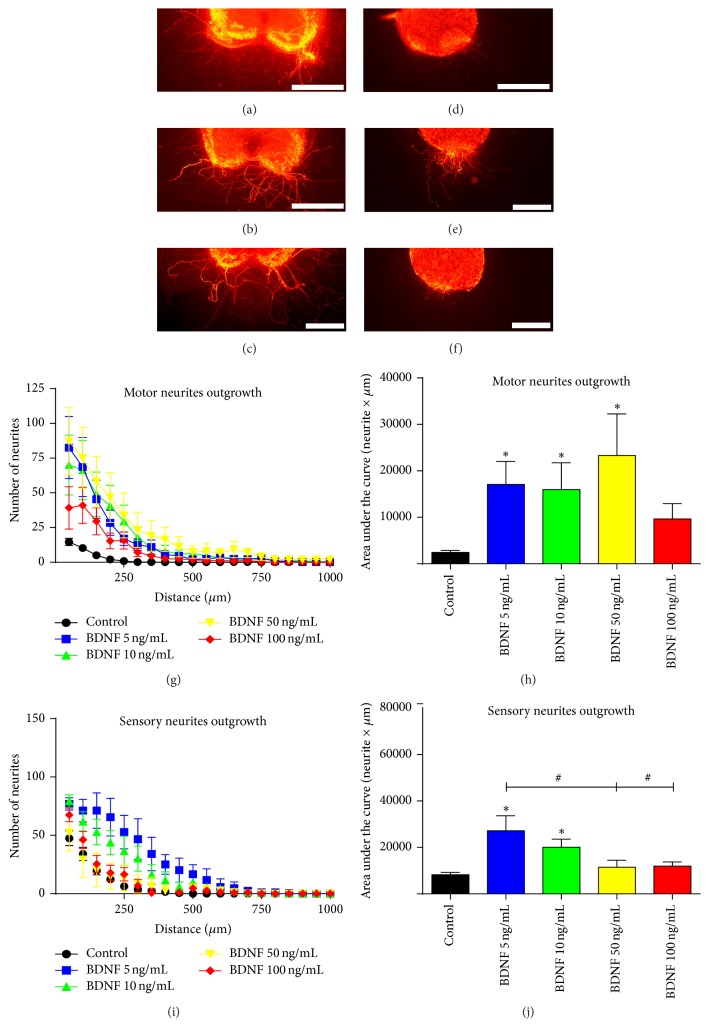
Representative images of RT97 stained neurites from spinal cord slices (a–c) and DRG neurons (d–f) cultured within a 3D collagen matrix alone (a, d) and with addition of 10 ng/mL (b, e) and 50 ng/mL of BDNF (c, f). Quantification of the number of neurites grown at increasing distance from the cord slices (g) and from DRG body (i) after the addition of different doses of BDNF. Plots of the quantified area under each curve from (g) and (i) graphs for motor (h) or sensory neurite outgrowth (j). Data expressed as mean ± SEM. ^*∗*^ *p* < 0.05 versus control; ^#^ *p* < 0.05. Scale bar: 200 *μ*m (a–f).

BDNF, NGF, NT-3, GDNF, and FGF-2 at different concentrations were tested to elucidate the optimal concentration of each NTF that is able to enhance either motor or sensory neurite outgrowth without affecting the other neuronal population. For that purpose, 3D organotypic cultures of SC slices and DRG were used (Figures 1 [Fig fig2]–3).

### 3.1. GDNF and FGF-2 Enhance Both Motor and Sensory Neurite Outgrowth

**Figure 4:**
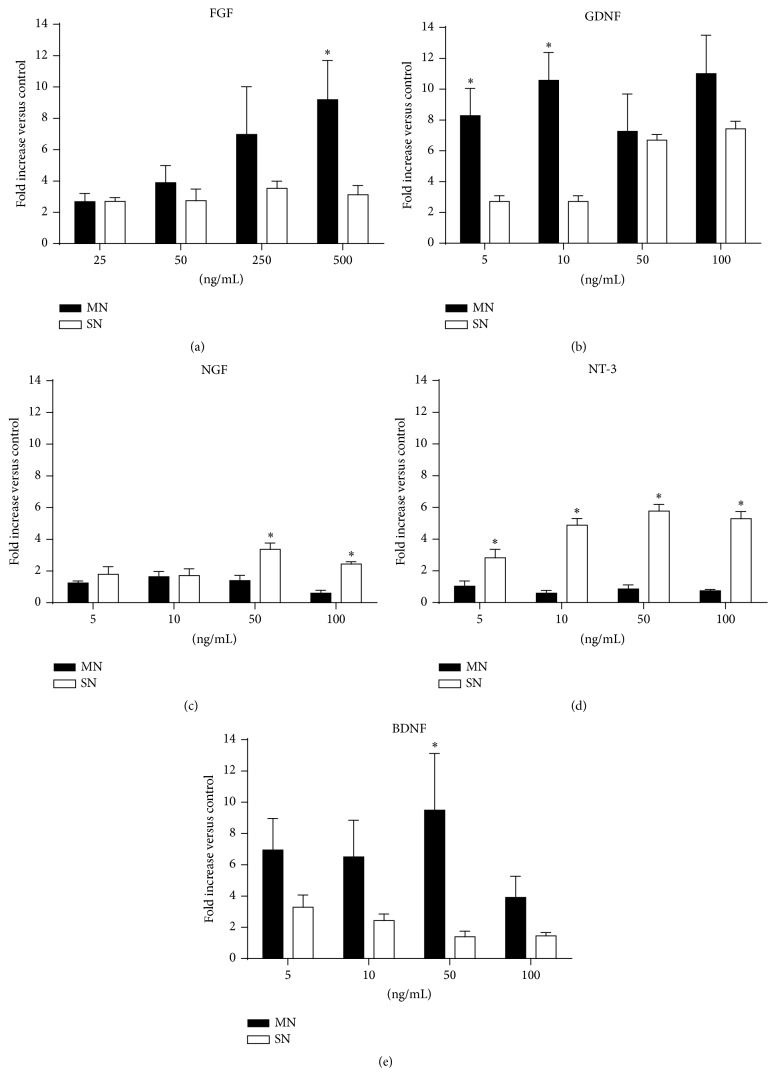
Histogram of the fold increase of the AUC for motor and sensory neurite outgrowth induced by FGF (a), GDNF (b), NGF (c), NT-3 (d), and BDNF (e) at the concentrations tested compared to the control collagen matrix. Data expressed as mean ± SEM. ^*∗*^ *p* < 0.05.

Almost all doses of both FGF-2 and GDNF increased motor and sensory neurite outgrowth with respect to the control substrate (Figure 1). Regarding motor neurite outgrowth, FGF-2 showed a progressive dose effect with the lowest concentrations not being able to reach significant differences versus the control group (Figures 1(g) and 1(i)), while GDNF enhanced motor neurite outgrowth with no dose-dependency as all the groups exhibit similar curves (Figures 1(h) and 1(j)). On the other hand, sensory neurites growth from DRG increased similarly at all the tested concentrations of FGF-2 (Figures 1(k) and 1(m)), while GDNF followed a dose-dependent increase showing the high doses even significantly larger AUC values than the low ones (Figure 1(n)). Comparatively, using the data of the AUC in each culture condition, FGF-2 promoted a maximum increase of about 9 and 3 times in neurite growth of both motor and sensory neurites, whereas GDNF induced maximal increase of 11 and 8 times baseline, respectively (Figures 4(a) and 4(b)).

These results in vitro reveal that neither GDNF nor FGF-2 show a preference for motor or sensory neuritogenesis, as both neuronal populations are enhanced at the different concentrations used. While GDNF has already been reported to promote neurite outgrowth of both populations, FGF-2 was described to preferentially enhance motor neurite outgrowth when measuring the length of longest neurites [15]. Since reliable measurements of neurite outgrowth analysis in organotypic cultures are complicated [21], we used a semiautomatic analysis that works as an adaption of the Sholl method [17] to improve accuracy and reproducibility as shown in other works [22, 23]. Thus, the differences in methods and variables to quantify neurite outgrowth in these studies may in fact explain some controversies. Hence, in accordance with our results, we discarded GDNF and FGF-2 for in vivo studies because of their lack of selective effect.

### 3.2. NGF and NT-3 Selectively Enhance Sensory Regeneration In Vitro but Not In Vivo

Observation of cultures of SC slices and DRG with NGF and NT-3 (Figures 2(a)–2(f)) revealed they were the most selective tested factors for sensory regeneration. Motor neurite outgrowth was not affected by NGF or NT-3 at any of the concentrations compared to the control cultures (Figures 2(g)–2(j)). In contrast, addition of NGF (Figures 2(k) and 2(m)) or NT-3 (Figures 2(l) and 2(n)) in the collagen matrix yielded more sensory neurites at different distances and larger values of the AUC in comparison with controls (Figures 2(m) and 2(n)), the 50 ng/mL dose being the one that showed the highest values for NGF and NT-3. In agreement with previous studies [24], NGF and NT-3 are able to promote exclusively sensory neurite outgrowth, without enhancing motor axon regeneration from SC slices (Figures 4(c) and 4(d)). This selective effect may be explained because motoneurons do not express TrkA receptor and after nerve injury the expression levels of TrkC receptor remain relatively unchanged [7].

**Figure 5:**
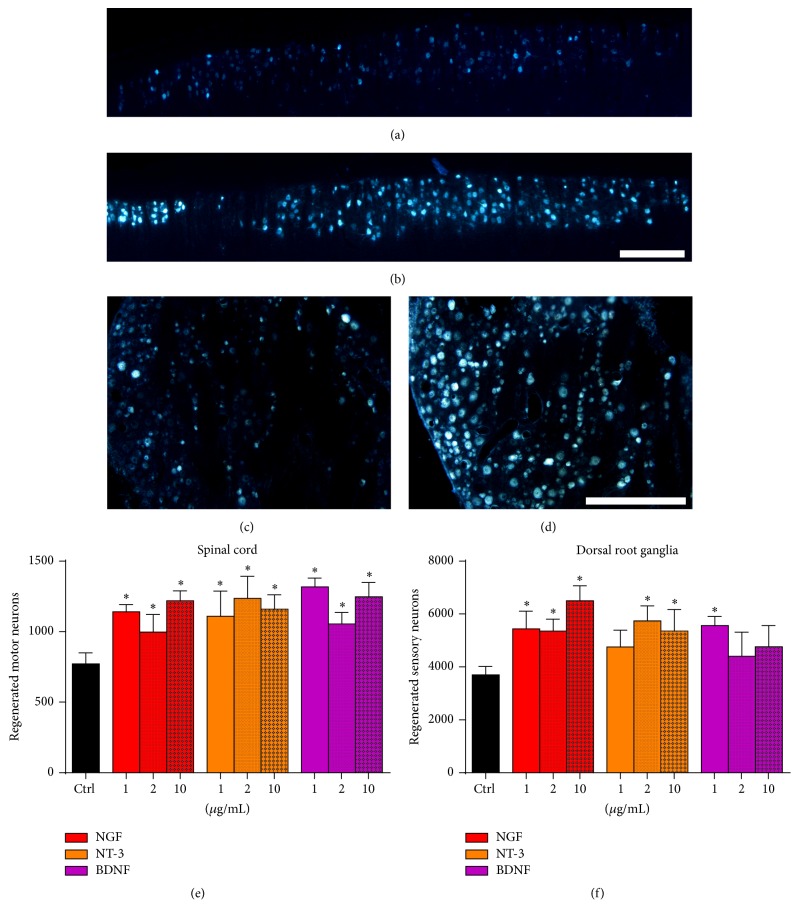
Representative micrographs of neurons retrolabeled with FG in the spinal cord (a, b) and DRG (c, d) growing in control conditions (a, c), with 10 *μ*g/mL of BDNF (b) and 10 *μ*g/mL of NGF (d). Histogram of the number of regenerated motor (e) and sensory (f) neurons after sciatic nerve section and conduit repair with NGF, NT-3, and BDNF at different doses. Data expressed as mean ± SEM. ^*∗*^ *p* < 0.05. Scale bar: 500 *μ*m (a–d).

Taking advantage of the differential promotion of sensory but not motor neurite outgrowth, these NTFs were tested in vivo in a model of nerve regeneration. 20 days after section of the sciatic nerve and tube repair, all the rats showed evidence of axonal regeneration, as judged by the retrograde labeling of motor (Figures 5(a) and 5(b)) and sensory (Figures 5(c) and 5(d)) regenerated neurons. Concerning motor axon regeneration, both NGF and NT-3 groups unexpectedly improved motor axon regeneration at all doses (Figure 5(e)). Indeed, although some studies have already described that NGF administration in a fibrin depot improves motor regeneration [25], the common belief is that NGF and NT-3 are prosensory NTFs [6]. Regarding sensory neurons, as expected, NGF and NT-3 groups showed a significantly increased number of regenerated neurons at all doses, except the lowest of NT-3 (1 *μ*g/mL) (Figure 5(f)).

Thus, in vivo results with NGF and NT-3 seem to contradict the in vitro observations where only sensory neurite outgrowth was improved. Differences between the in vitro and in vivo models should be first taken into account. Organotypic cultures are multicellular in vitro models, in which neurons remain embedded in contact with accompanying glial cells. Although Schwann cells and fibroblasts migrate outside the slice in organotypic cultures interacting with and giving structural support to the newborn neurites [26], the amount and activation of these cells are probably lower than in vivo conditions in which cells from the cut nerve stumps migrate inside the tube to stimulate nerve regeneration. In addition, the recruitment of hematogenous inflammatory cells that plays an important role during Wallerian degeneration in vivo is absent in vitro. Another important difference attains the role of extracellular matrix components. In our in vitro cultures, the matrix was made of collagen I only, whereas in vivo reactive Schwann cells and fibroblasts secrete several neurotropic molecules, including laminin and fibronectin [6]. It has been shown that neurite outgrowth in response to neurotrophins, such as NGF, is modulated by the composition and density of the extracellular matrix modifying the interactions with supporting Schwann cells [27].

After nerve damage, levels of NGF mRNA rise rapidly in the nonneuronal cells of the damaged nerve [28]. It is also known that NGF and other neurotrophins interact not only with axons but also with Schwann cells and fibroblasts within the regenerative microenvironment [29]. Taking into account that Schwann cells dedifferentiate after injury and change their phenotype to a proregenerative state [6, 30], secreting a variety of tropic and trophic molecules, both NGF and NT-3 might enhance axonal regeneration indirectly and indiscriminately by stimulating Schwann cell proliferation and release of other NTFs such as BDNF or GDNF [31, 32].

### 3.3. BDNF Selectively Enhances Motor Regeneration In Vitro and In Vivo

BDNF was the NTF showing the most selective effect on motoneuron regeneration in vitro (Figure 3). Addition of BDNF to the medium (Figures 3(b) and 3(c)) showed an increase in motor neurite outgrowth in comparison with control values (Figure 3(g)), the 50 ng/mL being the concentration that produced the highest values in terms of neurites length (Figure 3(g)) and AUC (Figure 3(h)). Interestingly, a higher concentration of 100 ng/mL did not promote neurite growth, suggesting a window of dose effect, in agreement with previous data [7, 9]. On the other hand, BDNF slightly enhanced sensory neurite outgrowth only at low concentrations of 5 and 10 ng/mL (Figures 3(e), 3(i), and 3(j)) while higher levels reverted this increase. These results suggest a preferential motor profile for BDNF with a high dose of BDNF (50 ng/mL) increasing about 10-fold the growth of motor neurites from SC slices but without effect on DRG neurite outgrowth (Figure 4(e)).

Following the in vitro results, we tested if BDNF could selectively promote motor axon regeneration in vivo after nerve section and tube repair. Quantification of retrolabeled neurons revealed that 20 days after surgery all the groups treated with BDNF had higher number of regenerated motor neurons than the control group (Figure 5(e)). In parallel to the in vitro results, BDNF only increased the number of regenerated sensory neurons at a low dose (1 *μ*g/mL) while higher doses gave similar result to the control group (Figure 5(f)).

The BDNF receptor TrkB is expressed constitutively in motoneurons and its levels increase after injury [7], while intact sensory neurons show low levels of TrkB, although its expression is also upregulated after injury [33]. Similarly, the endogenous expression of BDNF is also upregulated in the distal stump after nerve injury [7]. On the other hand, it has been suggested that BDNF upregulates TrkB expression at low doses [34], but high levels of this neurotrophin may downregulate this receptor in neuronal cells [35]. Thus, it seems that BDNF can modulate motor axon growth under a bimodal profile. Indeed, Boyd and Gordon demonstrated a biphasic effect in which low doses of exogenous BDNF increased the number of chronically axotomized motoneurons which regenerated their axons whereas high doses progressively reduced the number [9]. The low doses upregulate TrkB receptors and enhance both sensory and motor neurite growth promoting the expression of growth-associated genes such as tubulin or GAP-43 [36]. In contrast, high doses would downregulate TrkB receptors, minimizing the effect of BDNF or even promoting an inhibitory effect via activation of the p75 receptor [37]. The differential effect of the same doses on motor and sensory axonal regeneration might be explained because of differences in the expression of these receptors and their contribution to neurotrophin transport in motor and sensory neurons [38, 39]. On the other hand, the fact that the high dose of BDNF used in our study in vivo promoted motor axon regeneration can be explained because it is much lower than the doses shown to be inhibitory when applied by continuous infusion [9]. Other studies have proved that local release of BDNF increases the number of regenerated axons through grafts or conduits [40, 41], and a selective increase in the number of motor axons was noted with gene-induced BDNF overexpression [42]. At longer term after sciatic nerve injury and repair, administration of exogenous BDNF enhanced motor functional recovery in some studies [41, 43] but not in others [37, 42, 44]. Thus, the early administration of adequate concentrations of BDNF to axotomized motoneurons may be sufficient to sustain initial axon growth, but it may fail to support regeneration and target reinnervation for long time.

Schwann cells from motor and sensory nerve branches express different molecular markers that may contribute to the capacity of axons to specifically regenerate towards appropriate pathways. Particularly, Schwann cells associated with motor but not with sensory axons express the HNK1 carbohydrate epitope [45]. In addition, Schwann cells of sensory and motor nerves respond differently during denervation, by overexpressing different types of NTFs [46]. However, such differences tend to decline with time after injury, suggesting that the endogenous production of factors may not contribute enough to the sorting of different types of axons during nerve regeneration [6]. Interestingly, it has been shown that the upregulation of HNK-1 induced by electrical stimulation of the injured nerve is dependent on BDNF and its receptor TrkB [47], which are also increased by the electrical stimulation [48]. These observations suggest one mechanism by which exogenously modulating the local expression of BDNF may help to attract the regeneration of motor axons.

## 4. Conclusion

The results of this study provide a comparative analysis of the optimal doses for stimulating motor and sensory axonal regeneration both in vitro and in vivo for different NTFs (GDNF, FGF-2, NGF, NT-3, and BDNF). Optimal concentrations of GDNF and FGF-2 show the highest potentiation of both motor and sensory neuron regeneration. On the other hand, NGF and NT-3 show a selective enhancement of sensory neurite growth in vitro that is lost in our in vivo model. Finally, BDNF at selected doses selectively promotes motor axonal growth both in vitro and in vivo.
